# Global economic costs of herpetofauna invasions

**DOI:** 10.1038/s41598-022-15079-9

**Published:** 2022-07-28

**Authors:** Ismael Soto, Ross N. Cuthbert, Antonín Kouba, César Capinha, Anna Turbelin, Emma J. Hudgins, Christophe Diagne, Franck Courchamp, Phillip J. Haubrock

**Affiliations:** 1 Faculty of Fisheries and Protection of Waters, South Bohemian Research Center of Aquaculture and Biodiversity of Hydrocenoses, University of South Bohemia in České Budějovice, Zátiší 728/II, 389 25 Vodňany, Czech Republic; 2 School of Biological Sciences, Queen’s University Belfast, Belfast, BT9 5DL UK; 3 Centro de Estudos Geográficos, Instituto de Geografia e Ordenamento do Território-IGOT, Universidade de Lisboa, Rua Branca Edmée Marques, 1600-276 Lisbon, Portugal; 4 Laboratório Associado Terra, Lisbon, Portugal; 5 Université Paris-Saclay, CNRS, AgroParisTech, Ecologie Systématique Evolution, 91405 Orsay, France; 6 Department of Biology, Carleton University, Ottawa, Canada; 7 Department of River Ecology and Conservation, Senckenberg Research Institute and Natural History Museum Frankfurt, Gelnhausen, Germany

**Keywords:** Biodiversity, Environmental economics, Invasive species

## Abstract

Biological invasions by amphibian and reptile species (i.e. herpetofauna) are numerous and widespread, having caused severe impacts on ecosystems, the economy and human health. However, there remains no synthesised assessment of the economic costs of these invasions. Therefore, using the most comprehensive database on the economic costs of invasive alien species worldwide (InvaCost), we analyse the costs caused by invasive alien herpetofauna according to taxonomic, geographic, sectoral and temporal dimensions, as well as the types of these costs. The cost of invasive herpetofauna totaled at 17.0 billion US$ between 1986 and 2020, divided split into 6.3 billion US$ for amphibians, 10.4 billion US$ for reptiles and 334 million US$ for mixed classes. However, these costs were associated predominantly with only two species (brown tree snake *Boiga irregularis* and American bullfrog *Lithobates catesbeianus*), with 10.3 and 6.0 billion US$ in costs, respectively. Costs for the remaining 19 reported species were relatively minor (< 0.6 billion US$), and they were entirely unavailable for over 94% of known invasive herpetofauna worldwide. Also, costs were positively correlated with research effort, suggesting research biases towards well-known taxa. So far, costs have been dominated by predictions and extrapolations (79%), and thus empirical observations for impact were relatively scarce. The activity sector most affected by amphibians was authorities-stakeholders through management (> 99%), while for reptiles, impacts were reported mostly through damages to mixed sectors (65%). Geographically, Oceania and Pacific Islands recorded 63% of total costs, followed by Europe (35%) and North America (2%). Cost reports have generally increased over time but peaked between 2011 and 2015 for amphibians and 2006 to 2010 for reptiles. A greater effort in studying the costs of invasive herpetofauna is necessary for a more complete understanding of invasion impacts of these species. We emphasise the need for greater control and prevention policies concerning the spread of current and future invasive herpetofauna.

## Introduction

The transportation rate of alien species into new regions is unprecedented and accelerating with globalising trade and transport networks^1^. These often lead to invasions, with ecological impacts that include extinctions of native species and disruptions of ecosystem functioning^2^ through direct and indirect effects^3,4^. They also have major impacts on economic activities and human society—with high monetary costs to multiple economic sectors^5^, disruptions of livelihoods and loss of human health^6,7^ and welfare^8^. Synthesised assessments of the impacts of major taxonomic groups are sparse^9,10^ inhibiting management actions, assessment of biases, and the filling of knowledge gaps^11^.

In recent decades, invasive herpetofauna (i.e. amphibians and reptiles), have gained great interest on various platforms (e.g. social media) due to the numerous impacts they cause and the interest they generate from a wide range of sectors of society^12^. These impacts are present in different forms, such as ecological (i.e. extirpation or reduction of native species, e.g. decline of rare species via predation), evolutionary (i.e. genetic or morphological changes, e.g. hybridisation with natives species), epidemiological (i.e. spread of disease, e.g. chytridiomycosis caused by *Batrachochytrium dendrobatidis* and *Batrachochytrium salamandrivorans*^13^ and socio-economic (i.e. monetary and social impacts, e.g. through agricultural productivity declines or healthcare costs^14,15^) impacts. For example, the yellow-bellied slider *Trachemys scripta elegans* has degraded the functioning of ecosystems by altering abiotic characteristics and through the displacement of native species^16,17^; the cane toad *Rhinella marina* has led to morphological changes in the heads of native ranivorous snakes^18^, declines in native predators due to toxicity^19^, and caused substantial management expenditures^20^; the common coqui frog *Eleutherodactylus coqui* has led to declines in property values in areas infested due to its extremely loud mating song^21^; the brown tree snake *Boiga irregularis* has generated significant economic costs through island-wide power outages, resulting in a loss of US$ 4.5 million US$ per year^22^, as well as ecological impacts through the extirpation of most endemic birds via depredation^23^.

Yet, there are now more than 1000 known occurrences of alien reptile and amphibian species worldwide^24^ and the number of alien herpetofauna species has continued to increase in recent decades^1,25^, primarily due to the globalisation of human activity^26^ and other cultural or social factors^27,28^. Thus far, the primary introduction pathways for alien herpetofauna arise from unintentional and intentional transportation by humans, for example, via stowaways in cargo shipments or the nursery trade, and via the pet trade, religious releases, aquaculture or biocontrol programmes^24,25^. Furthermore, climate change is expected to increase the invasibility of regions such as Central and South America and central Africa to alien herpetofauna^29^.

Knowledge gaps regarding invasive herpetofauna are pervasive due to the lack of adequate data and the presence of geographic and taxonomic biases^30^. The filling of these knowledge gaps is crucial to incentivise a better development of invasion pathway regulatory policy and investment in control or biosecurity measures (e.g., trade of alien pets). In this context, the monetisation of the effects of invaders is a valuable option to raise societal awareness as well as motivate and justify policy actions by decision-makers^5^. The use of monetary values also allows highlighting of knowledge gaps among taxa using a common metric. The recent publication of the InvaCost database, where literature on the costs of alien species is compiled, standardised and described across a large number of indicators in a public form^31^, has increased interest in the synthesised study of invasive alien species monetary costs^32–35^.

Here, we quantify and synthesise, for the first time, the economic costs of alien herpetofauna worldwide, based on the current state-of-the-art in the published literature. We expect our results to reveal high costs, but also patterns and possible spatial biases that will inform the direction of future research: (i) we expect a minority of species will occupy the majority of the research effort and costs^36–38^; (ii) costs will be biased towards particular regions, as shown previously by other studies^5,36^; (iii) most economic costs will come from damage to primary sectors such as agriculture, due to these being commonly impacted by invasions^39^; and (iv) temporal trends will be characterised by substantially increasing costs over time, due to the increase in invasion rates worldwide^1^ and a growing number of pathways introducing herpetofauna.

## Results

### Economic costs by order and family

The total reported costs of the invasive herpetofauna exceeded 16.98 billion US$ between 1986 and 2020. We found that out of 280 invasive herpetofauna species recorded in the herpetofauna database^40^, 263 species did not have any economic impacts recorded in the InvaCost database (93.92%, Supplementary Table S1). For amphibians, we found that 75 of 81 species did not record a cost (92.59%), while 188 of 199 reptiles did not record a cost (94.47%). In particular, no cost was recorded for Caudata (Amphibia) and Crocodilia (Reptilia) entirely (Supplementary Table S1, Supplementary Appendix B). This lack of studies is magnified at the family level, where we only found costs for 5 out of 20 (25%) families of amphibians and 7 out of 37 (18.91%) families of reptiles (Supplementary Table S1). In addition, no economic cost was reported in Hylidae and Salamandridae, even though they are the most biodiverse families in terms of alien amphibian species^40^). Similarly, for the richest families of reptiles, Gekkonidae or Scincidae, no costs were reported.

### Cost among classes, reliability and implementation type

**Figure 1:**
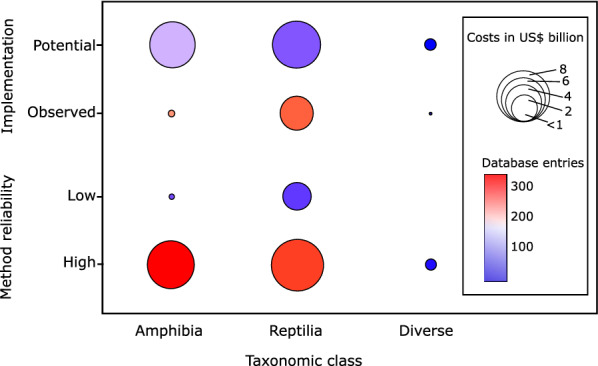
Global costs in US$ 2017 according to the type of implementation of cost (*Potential* vs. *Observed*) and method reliability (*Low* vs. *High*). The circle sizes indicate cost in US$ and the colours indicate the number of entries.

While amphibians contributed a total of 6.27 billion US$, nearly all costs originated from high reliability entries, amounting to more than 6.24 billion US$ (99.58%), compared to 26.08 million US$ (0.42%) in low reliability entries. In terms of implementation, only 70.56 million US$ came from observed costs (1.28% of costs), and 6.19 billion US$ from potential costs (98.72% of costs). For reptiles, 8.12 billion US$ (78.10%) of cost entries came from high reliability sources and 2.25 billion US$ (21.89%) from low reliability sources (Fig. 1). A share of 3.49 billion US$ (33.65%) of the total 10.37 billion US$ came from observed costs, while 6.88 billion US$ (66.35%) came from potential costs. For the diverse (i.e., mixed) class, all recorded costs (334.14 million US$) came from high reliability sources (Fig. 1). In addition, 327.01 million US$ (97.90%) came from potential costs and 7.13 million US$ from observed costs (2.10%).

### Cost among species and taxonomic group

**Figure 2:**
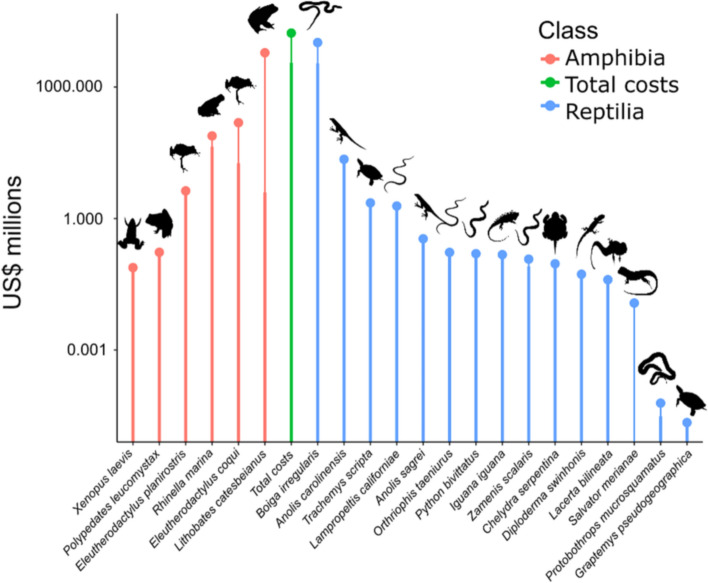
Cost per species for both classes. Red bars represent amphibians, while blue bars represent reptiles. The green bar represents the total cost of invasive herpetofauna. The wide, lower part of the bar represents the observed costs, while the thinner part represents the total costs (*Observed* plus *Potential* costs). Note that the *y*-axis is on a log_10_ scale. Animal silhouettes are from PhyloPic (phylopic.org).

The overall cost of invasive herpetofauna was due to a handful of species, which contributed the bulk of total costs (Fig. 2). For amphibians, the total cost of 6.27 billion US$ was divided among only six species, but a very large part of these costs came from a single species (American bullfrog, *Lithobates catesbeianus*) with a total cost of 6.04 billion US$ (96.33%, n = 63). The rest of the amphibian species accumulated less than 0.23 billion US$ (3.66%. n = 293).

For the reptiles, the total cost of 10.37 billion US$ was divided among 15 species, with the *B. irregularis* registering a total of 10.34 billion US$ (99.30%, n = 78), while the rest of reptilian species accumulated less than 0.03 billion US$ (0.70%). The species with the highest number of entries were *T. scripta* (n = 109), *B. irregularis* (n = 78) and the Green anole *Anolis carolinensis* (n = 60); the remaining species accounted for 91 entries in total.

For the amphibians, most of the observed costs belonged to *R. marina* (43.5 million US$; 61.42%; n = 155), followed by *E. coqui* with 18.30 million US$ (26.14%; n = 6). The remaining amphibian species (n = 5) recorded less than 8.3 million US$ (< 12.50%; n = 93).

For the reptiles, observed species-level costs totalled 3.49 billion US$, and *B. irregularis* dominated with 3.46 billion US$ (99.14%; n = 38), while the remaining species (n = 14) cumulated less than 28 million US$ (< 0.90%; n = 254).

We identified significantly greater (*W* = 4051.5, *p-value* ≤ 0.001) numbers of publications for the species with recorded costs in the InvaCost database compared to those that did not have any record of economic costs. Further, for those species with costs, we found a significant positive correlation between the total cost of the recorded species and the number of respective publications (*Adjusted R*^2^ = 0.26, *p-value* = 0.01) (Supplementary Fig. S1, Supplementary Appendix B). This relationship was also significantly positive in the case of observed costs only (*Adjusted R*^2^ = 0.16, *p-value* = 0.04; Supplementary Fig. S2, Supplementary Appendix B).

### Costs among activity sectors and types

For the amphibians, we found that the *Authorities-stakeholders* activity sector reported the highest economic costs (6.25 billion US$, 99.6%, n = 335) followed by *Public and social welfare* (20 million US$, 0.24%, n = 2) and *Agriculture* (8.98 million US$, 0.14%, n = 23), while the remaining impacted sectors recorded less than 728.88 thousand US$ together (< 0.01%; n = 6). Focusing on observed costs only, we found that the *Authorities-stakeholders* sector was still the most affected (48.04 million US$, 68.09%) by invasive amphibians, followed by *Public and social welfare* (13.27 million US$, 18.81%) and *Agriculture* (8.84 million US$, 12.52%). The remaining impacted sectors reported less than 397.12 thousand US$, < 0.56%).

For the reptiles, we found that the most impacted sector was *Diverse* (i.e. mixed; 6.75 billion US$, 65.1%, n = 37) followed by *Public and social welfare* (3.39 billion US$, 32.7%, n = 8) and *Authorities-stakeholders* (220 million US$, 2.17%, n = 278). The remaining impacted sectors recorded less than 415.38 thousand US$ together (< 0.003%, n = 15). This pattern was not repeated for observed costs considering invasive reptiles, with *Public and social welfare* (3.39 billion US$, 97.05%), followed by *Authorities-stakeholders* (99.44 million US$, 2.84%) and then the *Diverse* (3.10 million US$, 0.08%) sectors impacted most. The remaining impacted sectors reported less than 412.20 thousand US$ (< 0.01%).

**Figure 3:**
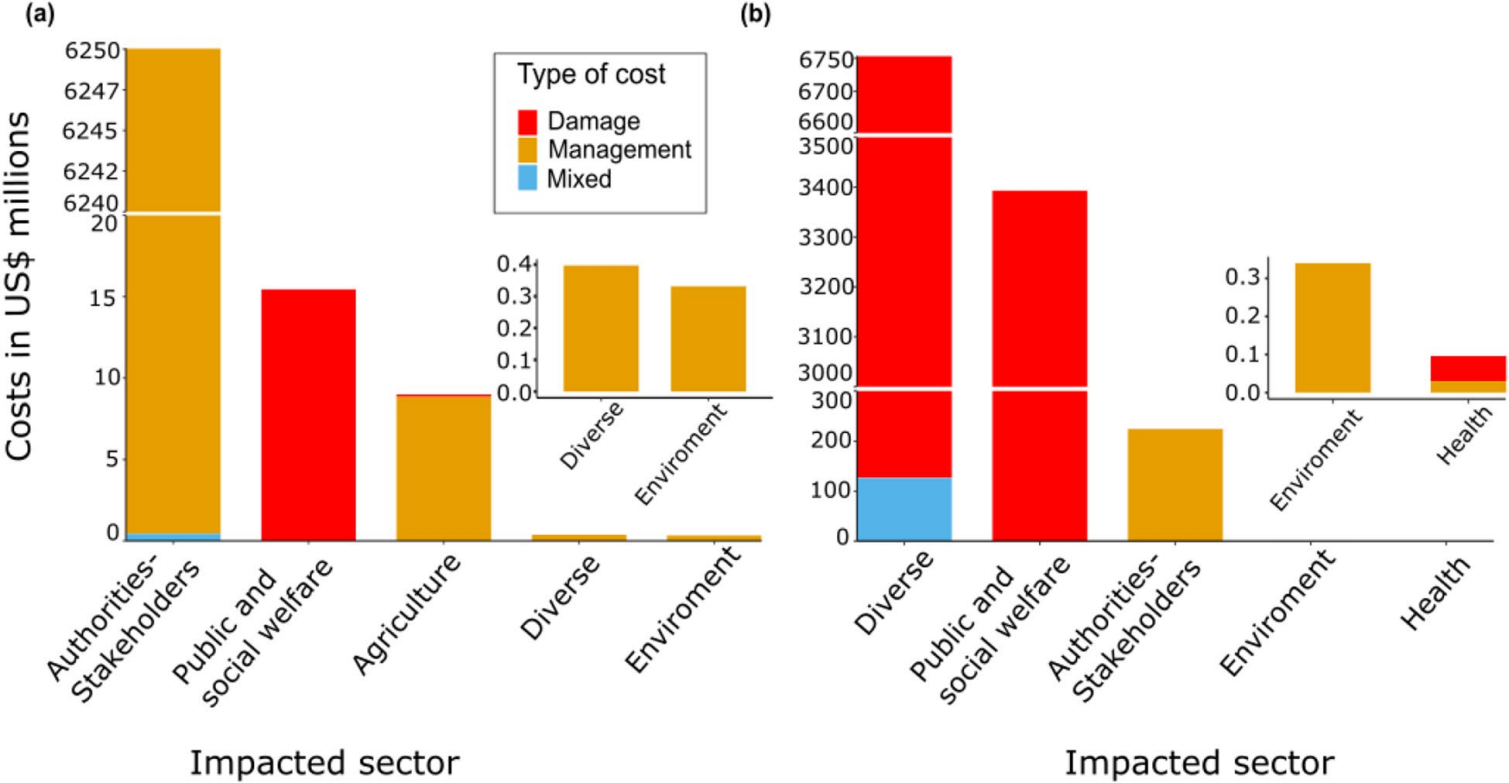
Total cost of alien (**a**) amphibian and (**b**) reptile species according to the sector impacted and their type of costs.

With respect to type of cost for amphibians, we found that the highest costs were for *Management* (6.25 billion US$, 99.7%, n = 385), followed by *Damage* (15.60 million US$, 0.25%, n = 3) and *Mixed* (441.91 thousand US$, 0.007%, n = 5) types. Focusing on *Observed* costs only, we found again that the highest costs were for *Management* (56.84 million US$, 80.55%) followed by *Damage* (13.27 million US$, 18.81%) and *Mixed* (441.98 thousand US$, 0.62%) types (Fig. 3).

For reptiles, we conversely found that the highest cost came from *Damage* (10.02 billion US$, 96.6%, n = 251), followed by *Management* (0.22 billion US$, 2.16%, n = 35) and *Mixed* (0.12 billion US$, 1.22%, n = 25) types. This pattern was repeated for *Observed* costs, with first *Damage* (3.39 billion US$, 97.16%), followed by *Management* (99.03 million US$, 2.83%) and *Mixed* (43.96 thousand US$, 0.001%) types (Fig. 3).

### Economic cost across geographic regions

**Figure 4:**
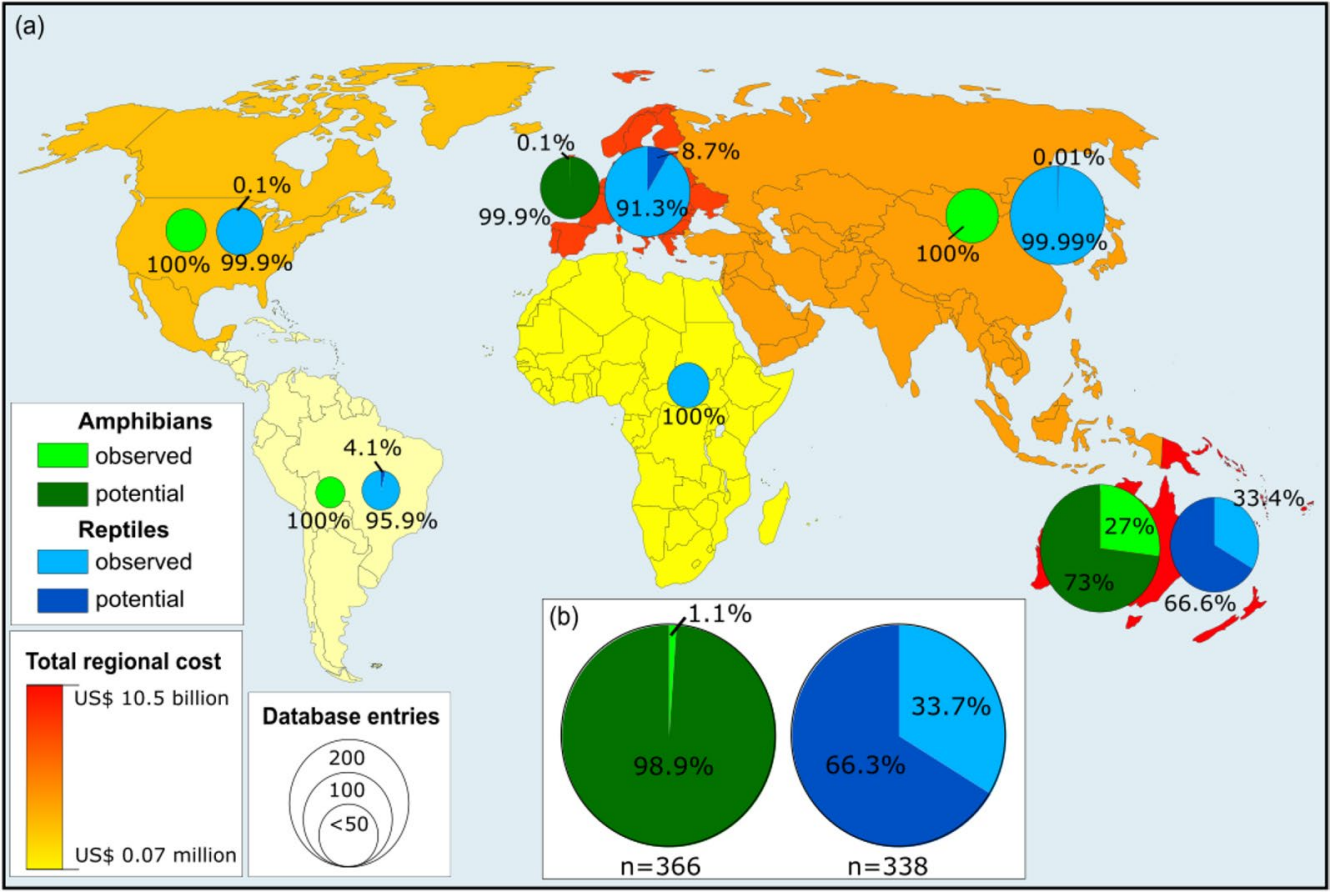
Geographical distribution of the economic costs of alien species, with the total regional cost of alien amphibians and reptiles (colour ramp), divided by Implementation (*Observed* vs. *Potential*) and scaled in size by the number of entries (**a**); and the total global cost divided by Implementation (**b**). Note that Africa has no recorded economic costs of alien amphibians.

Reported costs were strongly geographically clustered among classes. For the amphibians, we found that *Europe* incurred the highest economic costs (6.04 billion US$, 96.26%, n = 68) followed by *Oceania and Pacific Islands* (0.23 billion US$, 3.67%, n = 240) and diverse regions (3.25 million US$, 0.05%. n = 8). Of these costs, the majority was *Potential* (6.19 billion US$, 98.92%), being mostly in *Europe* (6.04 billion US$) and relatively little for *Oceania and Pacific Islands* (0.15 billion US$), while *Observed* costs were less than 72 million US$. The regions with the highest *Observed* costs were *Oceania and Pacific Islands* (62.14 million US$, 88.07%), followed by *Europe* (3.94 million US$, 5.59%) and *Diverse* (3.25 million US$, 4.61%). The remaining geographic areas recorded only a total of < 2 million US$ (0.01%, n = 50), split among Asia (1 million US$, 0.01%, n = 40), North America (0.03 million US$, < 0.001%, n = 9) and South America (0.002 million US$, < 0.001%, n = 1). Lastly, no economic costs of amphibians were recorded in Africa (Fig. 4).

For reptiles, we found that *Oceania and Pacific Islands* reported the highest costs (10.35 billion US$, 99.61%, n = 77), followed by *Asia* (25.00 million US$, 0.24%, n = 112) and *North America* (12.97 million US$, 0.12%, n = 18). Here, 66.24% came from *Potential* costs, with 6.86 billion US$ of the total, and 3.49 billion US$ (33.76%) came from *Observed* costs. The regions with the most costs observed were, again, *Oceania and Pacific Islands* (3.46 billion US$, 98.85%) followed by *Asia* (25.00 million US$, 0.71%) and *North America* (12.96 million US$, 0.37%). The remaining geographic areas recorded only a total of < 2 million US$ (0.01%, n = 126), split among Africa (1 million US$, 0.01%, n = 9), Europe (0.9 million US$, < 0.01%, n = 116) and South America (0.07 million US$, < 0.01%, n = 1) (Fig. 4).

### Temporal trends

**Figure 5:**
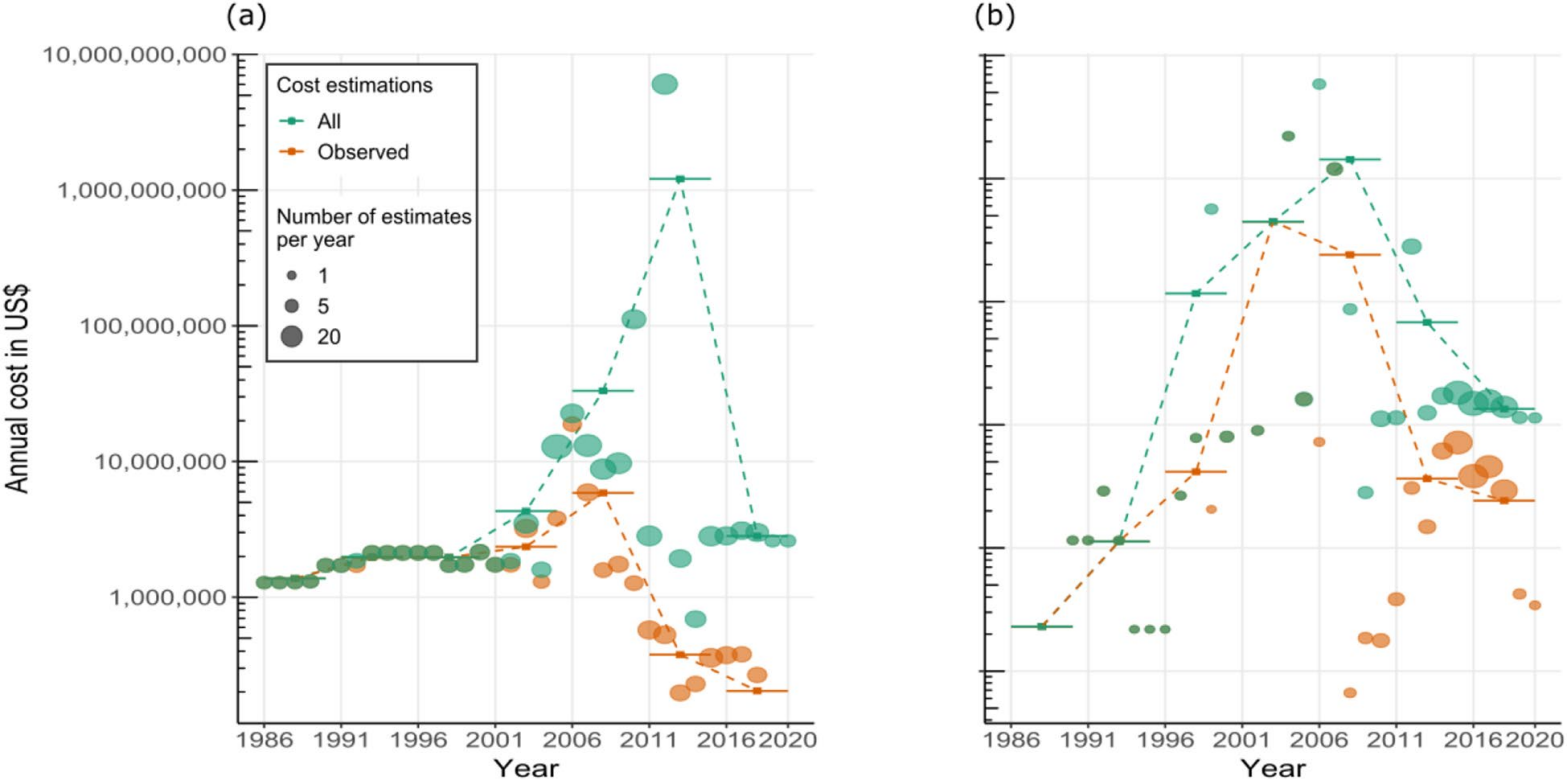
Total annual cost in US$ 2017 for (**a**) amphibians and (**b**) reptiles. The green colour represents all costs (*Observed* plus *Potential*), while the orange colour represents only *Observed* costs. The size of the circle indicates the number of estimates per year. Bars represent means for each 5-year period. Note that the y-axes are on log_10_ scales.

The total cost of invasive alien herpetofauna exceeded 16.98 billion US$ globally between 1986 and 2020, averaging US$ 485.21 million per year. The average annual cost of invasive amphibians totalled 179.29 million US$, with 1.21 billion US$ being the highest average annual cost in the period from 2011 to 2015 (Fig. 5a). The average annual cost of invasive reptiles was 296.37 million US$, with 1.43 billion US$, the highest average cost in the period from 2006 to 2010 (Fig. 5b).

Concerning only *Observed* costs for the amphibian class, we found 70.56 million US$ in total for the entire period, with the average annual cost being 2.01 million US$; the period in which the total average annual cost was the highest corresponds to 2006 to 2010 with 5.87 million US$. On the other hand, for the reptiles, an *Observed* cost of 3.49 billion US$ was found, corresponding to an average annual cost over the entire period of 99.88 million US$, with costs peaking between 2001 to 2005 with an average annual cost of 0.45 billion US$ (Fig. 5).

## Discussion

The present study found a total reported cost of global herpetofauna invasions of 16.98 billion US$ between 1986 and 2020. However, reported costs were principally due to just a few species, namely *B. irregularis*,* L. catesbeianus* and *R. marina* (~ 96%). There could thus be a positive feedback loop between research effort and cost reporting—with most research focused so far on a few high-profile species. Indeed, we found significantly greater numbers of papers concerning species with reported costs than those without reported costs. While InvaCost synthesises available cost information and allows it to be distinguished using various descriptors, we note the potential variability in underlying data that reflects methodological differences across the underlying literature. In addition, we acknowledge that the differences in research effort based on the number of papers for certain species, paired with a conceivable time lag in invasion and monetised impact reporting, may alter the total economic costs found in this study. These factors could make the total cost a conservative estimate, especially considering the gaps for those known invasive species that have few or no costs reported. Government policies may further influence research effort, resulting in certain widely distributed species receiving more attention from the scientific community. This corroborates earlier identifications of bias in studies of invasive alien herpetofauna^36^. While we acknowledge that not all invasive alien herpetofauna will have tangible monetary impacts, greater research effort is required to distinguish the true absence of economic cost from gaps in cost detection, especially for those taxa with the worst known ecological impacts^41^.

Most of the reported costs and entries were obtained from peer-reviewed literature or were deemed of high reliability. We note, however, that a binary classification of method reliability does not account for the variation of methods employed for varying taxa and regions in individual studies; they nevertheless provided a practical way of objectively assigning cost reliability. Further, these costs predominantly came from estimates and extrapolations, being evaluated as potential costs rather than empirically observed. This is the case for *L. catesbeianus potential costs* in Europe, which represent more than 95% of the total amphibian’s cost. Additionally, whereas the potential costs were higher, the number of entries was lower than the observed costs, indicating that potential cost entries tend to report much greater costs, which is to be expected since they often extrapolate costs from smaller to larger scales (e.g., from sub-national to national scales or for longer periods^42^.

We caution that potential costs could be overestimated or underestimated, and are accompanied with greater uncertainty than observed costs. For example, future actions by governments and stakeholders to prevent or contain the spread of alien species may reduce their potential damage costs, if initially estimated in the absence of effective management action (i.e. potential damage costs being reduced by the management measures taken after cost prediction). It is also possible that potential invasion costs were projected from one spatiotemporal unit to a much larger scale, but that the species then failed to spread, went bust or was eradicated, leading to an initial overestimation. For potential management costs, interventions can also become more efficient and less cost intensive as new protocols are developed, reducing the level of future costs than those predicted. However, in each of these scenarios, the reverse could occur, leading to an alternative underestimation of potential costs.

### Taxonomic costs across cost types

We found that most of the reported costs for amphibians are from management, while most other studies consistently show damage as the main source of costs, with values of 10–100 times higher than management^43,44^. This could indicate a massive underestimation of this cost type, although this pattern is not found in reptiles. Especially surprising is the low reported cost from Burmese python *Python bivittatus* considering the large number of impacts generated in the USA by the high predation capacity of this species, which has caused the decline of native species^45^, as well as great effort in the country to control its populations^46^. Yet, many of these ecological impacts are not monetised or available as they are not in the InvaCost database, and all recorded economic costs generated by *P. bivittatus* thus far are from a single eradication program targeting this species in Florida^47^. In addition, there is a possible cost bias as our study range is limited to 1986–2020, while some of the most notorious species were introduced long before that [e.g. *R. marina* were introduced in 1936 in Australia^48^].

The lack of reported costs found for the vast majority of reported alien species corroborates other studies at various spatial and taxonomic scales, including for the United Kingdom^37^, Singapore and Southeast Asia^35^, fishes^49^, and ants^50^, among others. Concerning sectors of economic activity for amphibians, most costs impacted authorities-stakeholders. The vast majority of these costs were recorded from post-invasion management actions (e.g. control or eradication), and so the improved investment in and development of prevention measures could reduce future economic costs from invasions by reducing needs for long-term management and the scale of incurred damage. A more diverse pattern was found for reptiles among sectors, emphasising a large amount of damage from these species across various sectors, which could again be mitigated by more timely management.

### Gaps in economic costs of herpetofauna invasions

Costs of herpetofauna invasions were of a similar or greater magnitude to other taxa, despite knowledge gaps, such as rodents (3.28 billion US$^5^, freshwater crayfish (120.50 million US$^51^ and the cost of all marine species (3.6 billion US$^34^. Alarmingly, we found a lack of economic costs for alien amphibians with substantial ecological impacts based on the Environmental Impact Classification for Alien Taxa (EICAT) and concomitant “Massive” effects on recipient communities, such as the tiger salamander (*Ambystoma tigrinum*) and Italian pool frog (*Pelophylax bergeri*)^41^. However, the impacts of these species appear to be characterised by hybridization^52,53^, and therefore these impacts are difficult to quantify economically due to their ecological nature. Regarding reptiles, we observed that three of the five most notorious aliens (*A. carolinensis*, *Anolis sagrei* and *B. irregularis*^54^) had reported economic costs, but we did not find economic costs for the remaining two: the curious skink (*Carlia ailanpalai*) and common house gecko (*Hemidactylus frenatus*) in the InvaCost database. To date, there is no assessment of the effects of reptiles in recipient ecosystems based on EICAT, but we hope that addressing for the first time the economic costs for these taxa will motivate their ecological assessment in the future.

### Geographical distribution of economic costs

While taxonomic gaps were pervasive across socio-economic sectors, these were coupled with a similar lack of data for geographic regions (although there may also be bias in geographical economic costs due to factors such as language^55^. The regions most studied were Oceania and Pacific Islands, Europe and Asia, while the least studied were Africa, Central America and South America. However, this difference may also arise from the number of alien species established in each region and research capacity in general. Most of the studies in Oceania and Pacific Islands corresponded to the effects of *R. marina* in Australia^56^ and *B. irregularis* in Hawaii and Guam^57^. Surprisingly, and in contrast to other studies^34,58^, a small number of studies from North America were observed. North American (especially in the USA) studies pioneered early works in economic costs estimations for invasive alien species^59^, promoting further research in the last few decades that resulted in burgeoning reported economic impacts from invasions there^54,60^.

Between the two classes, we found that the bulk of the costs were recorded from reptiles, especially in Oceania and Pacific Islands, except for Europe where amphibian costs were dominant for both total and observed costs. In particular, a large part of these European costs stems from the extrapolation of the economic impact of *L. catesbeianus* in Germany^61^. For amphibians, no costs were recorded in Africa, although there are several species of invasive alien amphibians in this region that cause problems, such as guttural toad *Sclerophrys gutturalis*, generating a direct impact on the local economy by the displacement of native species and species eradication programmes^62^. Africa is also the geographic region with the lowest number of total papers on alien herpetofauna, which based on our analysis of the relationship found between the number of studies and the costs of invasive species, could explain the lack of economic costs found in Africa^36^. For reptiles, Europe was one of the regions with the lowest recorded costs, alongside Central and South America. However, this could change in future years since the Central and South American region is expected to be most affected by herpetofauna invasions^29^.

In addition, costs from *B. irregularis* here were centred on studies from the Pacific islands (Hawaii, Guam and Northern Mariana Islands). Island ecosystems are regarded as more vulnerable to invasion by alien species and their impacts^62^. They can incur economic costs through a different suite of sectors and species than mainland areas^64^. Islands are substantially more biodiverse than mainlands^65^. While ecological impacts can be challenging to monetise, alien species there generate high rates of extinction of native species in groups such as vertebrates and plants^66^, which can have high intrinsic or cultural value^67^. Nonetheless, our study may help develop more effective policies to reduce these future economic costs.

### Temporal trends

Analysis of the cost trend between 1986 and 2020 revealed similar patterns for both classes. For amphibians, the cost increased until 2006, when it declined steeply. For the reptiles, the cost increased until 2006–2010, when it declined steeply until the final years of this study. Primarily, these trends likely relate to the delay in reporting impacts^5^, as well as the possibility that there is a lag phase in the impacts generated by these species, which can take decades^68^. Indeed, the range in which the greatest costs of amphibians was detected coincides with the years with the highest number of entries, with a total of 105 entries between 2006 to 2010, while recent years' entries record a much lower amount, with only 46 entries between 2016 to 2020. This pattern was not seen in reptiles, where in the period with the highest number of costs from 2011 to 2015, only 40 entries were reported. On the contrary, 149 entries were reported from 2016 to 2020. We, therefore, suggest that the presented costs over time for herpetofauna invasions have been heavily influenced by the evolution of the costs of the three costliest species (*B. irregularis*, *L. catesbeianus* and *R. marina*) and the timing of underlying studies.

### Future directions

This study presents the first global synthesis of the costs of invasive herpetofauna based on the current state-of-the-art in the InvaCost database. While costs are high, they are likely severely underestimated due to knowledge gaps, with costs focused on only a few taxa, regions and limited to recent decades^32^. Furthermore, because invasion rates are expected to increase in the future^69^, we expect this increase to be followed by a rise in economic costs. Yet, it could be reduced by investing in management, especially in the prevention of the arrival of alien species and early detection measures^70,71^. While research interest and economic impacts of biological invasions are globally increasing, their costs have been shown to grow faster than the relative number of reports of costs^72^. For herpetofauna, because these trends were driven by specific species or taxonomic groups, it can be expected that these will be prioritised, if neglecting wider data gaps. However, future research priorities should also be based on the recognition of data gaps, as that would result in improved research efforts to address the lack of data (e.g. on damages) and focus not only on those sites with the highest records of economic costs by invasive herpetofauna (e.g. Guam for reptiles and Australia for amphibians) but also on the monetisation of the costs generated by the ecological degradation by other herpetofauna species (e.g. *A. tigrinum* and *P. bergeri*).

Further, the main pathway of invasion of alien herpetofauna is the pet trade^73^. For amphibians, it is expected that future trade will not differ from the current patterning, where some of the species that are expected to still be traded widely are the yellow cururu toad *Rhinella icterica*, Müller's platanna *Xenopus muelleri* or Woodhouse's toad *Anaxyrus woodhousii*^74^. In contrast, the reptile pet trade is expected to change its most traded species in the future, expectedly resulting in new economic impacts to potentially different sectors. While *I. iguana*, *P*. *bivittatus* and *T. scripta* have declined the most in popularity, other species such as blue-tongued lizards *Tiliqua* spp., crested geckos *Correlophus ciliatus*, and ball pythons *Python regius* are expected to increase with a growing economic cost^36^. For this reason, the continuous updating of lists of invasive (or potentially invasive) species found in the pet trade could result in large economic savings for governments and stakeholders^75^.

Because of a high reliance so far on predictions and extrapolations, we urge further work to quantify costs actually incurred by invasive herpetofauna, which could in turn inform more robust predictive efforts and cost–benefit analyses of these animals for managers. In addition, pre-invasion management techniques could save a large economic burden for governments and stakeholders from invasive species damages and long-term management^70,76^. Given the high context-dependence in invasion science, whereby the impacts generated may vary depending on the area invaded, habitat type and socio-economic sector, we also recommend conducting assessments at more granular spatial and taxonomic scales^77^. We emphasise the importance of the development of policies by the responsible authorities and governments to prevent the arrival of alien species since effective prevention will decrease future expenses^70^.

## Materials and methods

### Data collection

To estimate the costs of invasive alien reptiles and amphibians worldwide, we used the most recent version (4.0; released in June 2021) of the InvaCost database (https://doi.org/10.6084/m9.figshare.12668570) at the time of writing, per the “getInvaCostVersion” function of the invacost R package^31,78^. This version of the database contains a total of 13,123 cost entries, extracted primarily from four different sources: Web of Science (https://webofknowledge.com/), Google Scholar (https://scholar.google.com/), Google search engine (https://www.google.com/) and biological invasion experts, that were contacted as part of this collection and identification process, to gather additional data. The collection and collation of data from these sources were based upon systematic search strings used to identify the costs of biological invasions^5^. The InvaCost database contains entries reported in 15 languages in a sufficiently detailed manner to analyse the cost of invasive alien species^55^.

Presently, InvaCost is the most complete public database on the economic costs of invasive alien species and whose methodology allows standardising costs over time and across countries. This database is continuously updated either by corrections (e.g. duplicate entries) or by adding new data. The reliability of the data is therefore reliant on the underlying reporting material. InvaCost, however, does not include benefits quantitatively. While these data were also screened according to their method reliability in an objective manner, we could not capture the full spectrum of underlying approaches to cost estimation in a holistic way. Each entry was converted into 2017 US dollars to standardise the cost values, making costs directly comparable. In addition, each entry has 65 descriptor variables, including, for example, information on species’ taxonomy, the type of ecosystem invaded and the type of economic impact caused. The data in InvaCost were carefully screened to minimise spatial overlaps, e.g. at local and national scales over time.

### Data processing

**Figure 6:**
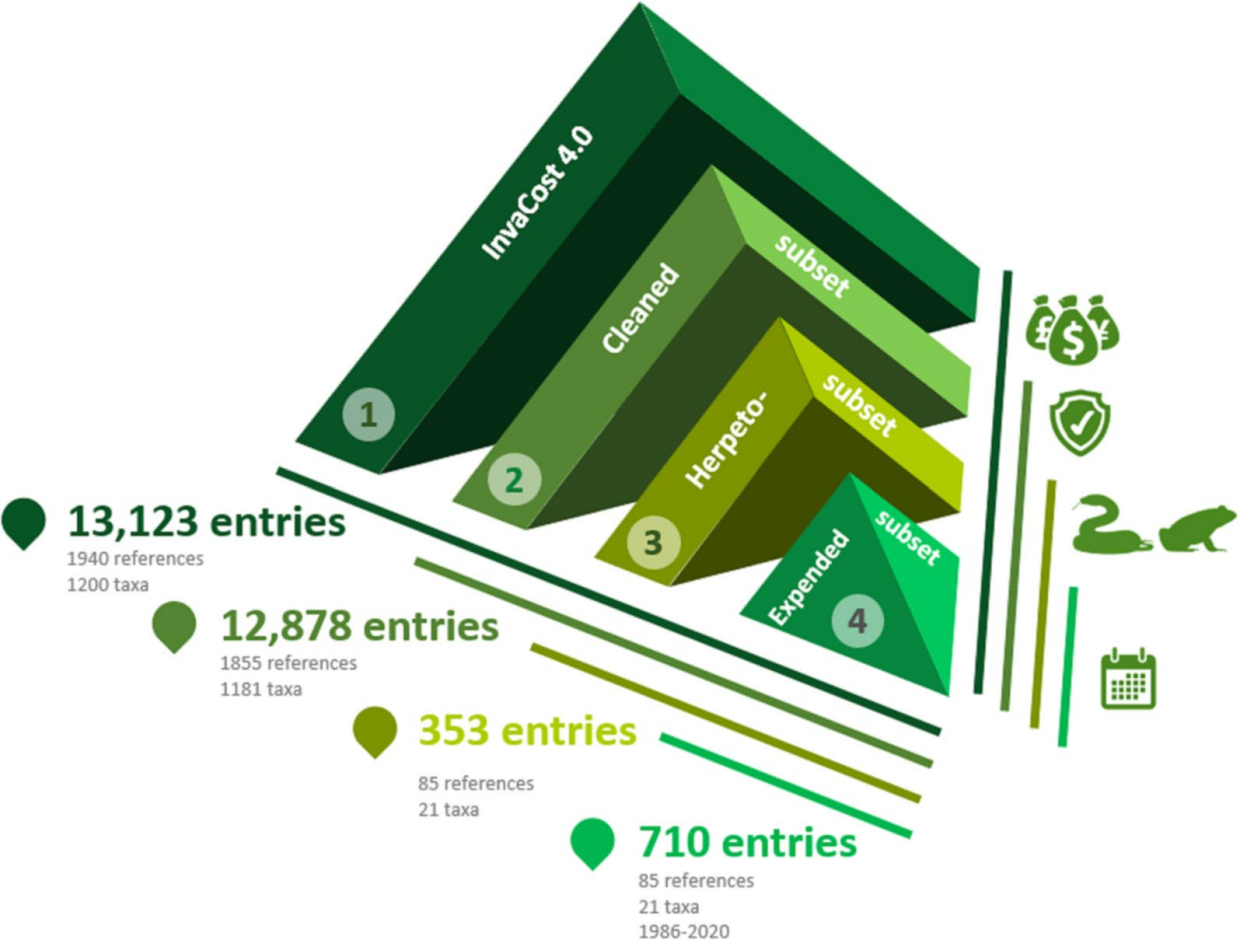
Diagram of the data selection and processing workflow.

We performed a series of steps before proceeding with the analysis of the data (Fig. 6). First, we refined the database by eliminating data with no information on starting and ending years (i.e. incomplete temporal scale; see later) and no monetised cost in the column “Cost_estimate_per_year_2017_USD_exchange_rate”. Next, we filtered the database to include only the “Amphibia” and “Reptilia” classes (column: “Class”, n = 354). Entries belonging to the class “Amphibia/Reptilia” and “Reptilia/Amphibia” were included in a new “Class” group (“Diverse”, n = 6). On the other hand, an entry recorded as “Amphibia/Arthropoda/Mammalia/Diverse” was excluded from the study due to the impossibility of separating the unique costs of amphibians. The total costs of the alien species were calculated with the function “summarizeCosts” of the invacost package^78^. This function summarises the cumulative costs and average annual costs of invasive alien species and splits the total costs evenly into annual cost estimates reflecting the respective period over which the cost was incurred, without inflating the cost estimates^78^.

This final dataset contained 353 entries which were all homogenised and converted on an annual basis, whereby the total cost for each estimate and each species was divided by the duration time of each cost entry. This process of ‘expanding’ the dataset was performed by the function “expandYearlyCosts'' of the invacost R package^78^. This function expands the estimated costs over the years they occurred or were estimated to have occurred. This means that one cost entry that reports a total cost of 100 million over 10 years, for example, will be transformed into ten cost entries with an annual cost of 10 million dollars each. The duration of the study was determined from the columns “Probable_starting_year_adjusted” for the start of the impact and “Probable_ending_year_adjusted” for the end of the impact. All analyses were carried out on this expanded database to reflect the duration of the costs according to each entry. Our final, expanded dataset had 710 entries from 1986 to 2020, thus covering 35 years.

### Cost descriptors

The cumulative costs of invasive alien species were aggregated according to the following descriptor variables:

I. *Method reliability *Parameter used to indicate the degree of reliability of the estimation of costs. A cost is classified as *High reliability* if it is reported from official reports or peer-reviewed articles, and if not, whether the estimation method is reproducible; if these criteria are not met, the estimate will be categorised as being of *Low reliability*.
II. *Implementation *Indicates whether the cost estimate has been produced directly in the invaded study area (*Observed; i.e.* cost actually incurred), or whether it is instead a predicted or extrapolated cost derived from the distribution area of alien species elsewhere, i.e., a cost not incurred but is predicted to occur over time and/or space expected (*Potential*). Examples of potential costs are the extrapolation of economic impacts due to the loss of tourist revenue from the presence of *R. marina* in Australia^19^, or the cost predicted for the removal of *B. irregularis* in Guam^57^. Further, to avoid the overestimation of extrapolated costs, the InvaCost database followed a strict selection and classification of costs, notably excluding previous potential costs that were later incurred and listed as observed costs.
III. S*pecies* This descriptor shows the name of the species responsible for the incurred costscost totals per species. In addition, entries that contained more than one species that could not be distinguished (column: “Species”) were added to the “Species” category “*Diverse*” (n = 44).
IV. *Impacted sector* market and/or activity sector affected by the costs of invasive herpetofauna, (e.g. *Agriculture*, *Health*, *Public and social welfare*). All entries with more than one category were pooled into “*Diverse*”.
V. *Type of costs* Type of cost generated by the invasion: *Damage* (e.g. yield loss, health injury), *Management* (e.g. control, research) and *Mixed* (indistinguishable damage and management components).
VI. *Geographic region *Refers to the continental region where the cost of alien species invasion was recorded. We also made several changes to the descriptor variable “Geographic_region” to homogenise the sub-group. These changes were the joining of “Pacific Islands” and “Pacific Islands/Oceania” into “*Oceania and Pacific Islands*”, and “Central America” and “South America” into “*Central/South America*” and “Oceania/South America'' into *''Diverse*”.

We also examined the proportion of invasive alien species for which costs were reported using a database reporting alien herpetofauna worldwide, separately by order and family^26,40^. To our knowledge, this is the most complete database published on invasive herpetofauna records, whereby each entry represents the occurrence of an alien established species in a country, federal state or biogeographically-separate island or archipelago.

Furthermore, we examined whether species with costs were more studied than those without costs, and correlated the magnitude of costs with the number of studies (Supplementary Appendix A). We used an unpaired two-sample Wilcoxon test with an alpha level of 0.05 to compare study numbers between species with (i.e., in InvaCost) and without (i.e., not in InvaCcost) costs. We then assessed whether the magnitude of total and observed costs of invasive alien species in InvaCost was correlated to their respective numbers of associated papers through linear regressions. We assessed the normality of residuals using a Shapiro–Wilks test and performed a logarithmic transformation [log10(x + 1)], as costs did not comply with this assumption to normalise the variables.

To assess the trend of alien reptilian and amphibian species costs, we considered 5-year intervals ranging from 1986 to 2020 due to data availability. We used the “summarizeCosts” function of the invacost package^75^ to estimate the total costs of alien species per year and the mean for each 5-year period, considering amphibians and reptiles separately.

## Supplementary Information

Supplementary Information.
