# Supplementary material for: Global economic costs of herpetofauna invasions
Source: Sci Rep. 2022 Jul 28;12:10829. doi: 10.1038/s41598-022-15079-9 (PMC9334389; doi:10.1038/s41598-022-15079-9)
Supplement: Supplementary file 1 — Supplementary Information. [file 41598_2022_15079_MOESM1_ESM.docx]

**Global economic costs of herpetofauna invasions**

Ismael Soto^1^; Ross N. Cuthbert^2^; Antonín Kouba^1^; César Capinha^3,4^; Anna Turbelin^5^; Emma J. Hudgins^6^; Christophe Diagne^5^; Franck Courchamp^5^ & Phillip J. Haubrock^1,7^

^1^ University of South Bohemia in České Budějovice, Faculty of Fisheries and Protection of Waters, South Bohemian Research Center of Aquaculture and Biodiversity of Hydrocenoses, Zátiší 728/II, 389 25 Vodňany, Czech Republic

^2^ School of Biological Sciences, Queen’s University Belfast, BT9 5DL Belfast, UK

^3^ Centro de Estudos Geográficos, Instituto de Geografia e Ordenamento do Território - IGOT, Universidade de Lisboa, Rua Branca Edmée Marques, 1600-276 Lisboa, Portugal

^4^ Laboratório Associado Terra, Portugal

^5^ Université Paris-Saclay, CNRS, AgroParisTech, Ecologie Systématique Evolution, 91405, Orsay, France

^6^ Department of Biology, Carleton University, Ottawa, Canada

^7^ Senckenberg Research Institute and Natural History Museum Frankfurt, Department of River Ecology and Conservation, Gelnhausen, Germany.

Correspondence: phillip.haubrock@senckenberg.de

**Appendix A. Supplementary material**

A Web of Science search was performed in July 2021 which included the following string: latin name of *species name* AND (non-native OR nonnative OR non-indigenous OR nonindigenous OR alien OR exotic OR introduced OR colonizing OR invasive), thus combining each species’ name with a synonym of alien. Studies that reported an economic cost (i.e. overlap with Invacost) were removed from the total number for the corresponding species to reduce potential overestimation in the total number of studies.

**Appendix B. Supplementary material**

**Table S1:** Summary of species without costs found at the order and family levels for herpetofauna invasions in InvaCost compared to known aliens from Capinha et al. (2017).

| **Order** | **Class** | **Species without costs** | **Percentage without costs** | **Observed costs^ϴ^** | **Total costs^ϕ^** |
| --- | --- | --- | --- | --- | --- |
| Anura | Amphibia | 62/68 | 91.17 | 1.97 | 36.95 |
| Caudata | Amphibia | 13/13 | 100 | NR | NR |
| Squamata | Reptilia | 155/163 | 95.09 | 97.7 | 61.05 |
| Testudines | Reptilia | 32/35 | 91.42 | 0.07 | 0.01 |
| Crocodilia | Reptilia | 1/1 | 100 | NR | NR |
| Diverse | Amphibia/Reptilia | 0/0 | 0 | 0.25 | 1.97 |
| **Family** | | | |  |  |
| Bufonidae | Amphibia | 6/7 | 88.7 | 1.21 | 0.45 |
| Eleutherodactylidae | Amphibia | 4/6 | 66.66 | 0.63 | 0.92 |
| Rhacophoridae | Amphibia | 1/2 | 50 | 0.004 | 0.001 |
| Pipidae | Amphibia | 0/1 | 0 | 0.002 | 0.0004 |
| Ranidae | Amphibia | 23/24 | 95.83 | 0.11 | 35.6 |
| Alytae | Amphibia | 2/2 | 100 | NR | NR |
| Ambystomatidae | Amphibia | 1/1 | 100 | NR | NR |
| Bombinatoridae | Amphibia | 1/1 | 100 | NR | NR |
| Cryptobranchidae | Amphibia | 1/1 | 100 | NR | NR |
| Dendrobatidae | Amphibia | 1/1 | 100 | NR | NR |
| Dicroglossidae | Amphibia | 4/4 | 100 | NR | NR |
| Hylidae | Amphibia | 10/10 | 100 | NR | NR |
| Leptodactylidae | Amphibia | 1/1 | 100 | NR | NR |
| Microhylidae | Amphibia | 3/3 | 100 | NR | NR |
| Pelodryadidae | Amphibia | 4/4 | 100 | NR | NR |
| Plethodontidae | Amphibia | 4/4 | 100 | NR | NR |
| Proteidae | Amphibia | 2/2 | 100 | NR | NR |
| Ptychadenidae | Amphibia | 1/1 | 100 | NR | NR |
| Pyxicephalidae | Amphibia | 1/1 | 100 | NR | NR |
| Salamandridae | Amphibia | 5/5 | 100 | NR | NR |
| Chelydridae | Reptilia | 1/2 | 50 | 0.002 | 0.0005 |
| Colubridae | Reptilia | 17/20 | 85 | 97.05 | 60.9 |
| Dactyloidae | Reptilia | 17/19 | 89.47 | 0.63 | 0.13 |
| Emydidae | Reptilia | 10/12 | 83.33 | 0.06 | 0.01 |
| Iguanidae | Reptilia | 5/6 | 83.33 | 0.004 | 0.0008 |
| Lacertidae | Reptilia | 9/10 | 90 | 0.001 | 0.0002 |
| Teiidae | Reptilia | 6/7 | 85.71 | NR | 0.00006 |
| Pythonidae | Reptilia | 3/3 | 100 | NR | NR |
| Viperidae | Reptilia | 2/2 | 100 | NR | NR |
| Agamidae | Reptilia | 6/6 | 100 | NR | NR |
| Acrochordidae | Reptilia | 1/1 | 100 | NR | NR |
| Alligatoridae | Reptilia | 1/1 | 100 | NR | NR |
| Amphisbaenidae | Reptilia | 1/1 | 100 | NR | NR |
| Anguidae | Reptilia | 1/1 | 100 | NR | NR |
| Blanidae | Reptilia | 1/1 | 100 | NR | NR |
| Boidae | Reptilia | 2/2 | 100 | NR | NR |
| Chamaeleonidae | Reptilia | 7/7 | 100 | NR | NR |
| Chelidae | Reptilia | 1/1 | 100 | NR | NR |
| Corytophanidae | Reptilia | 1/1 | 100 | NR | NR |
| Crotaphytidae | Reptilia | 1/1 | 100 | NR | NR |
| Gekkonidae | Reptilia | 34/34 | 100 | NR | NR |
| Geoemydidae | Reptilia | 6/6 | 100 | NR | NR |
| Gymnophthalmidae | Reptilia | 1/1 | 100 | NR | NR |
| Lamprophiidae | Reptilia | 2/2 | 100 | NR | NR |
| Leiocephalidae | Reptilia | 2/2 | 100 | NR | NR |
| Leptotyphlopidae | Reptilia | 1/1 | 100 | NR | NR |
| Liolaemidae | Reptilia | 1/1 | 100 | NR | NR |
| Pareidae | Reptilia | 1/1 | 100 | NR | NR |
| Pelomedusidae | Reptilia | 2/2 | 100 | NR | NR |
| Phrynosomatidae | Reptilia | 2/2 | 100 | NR | NR |
| Phyllodactylidae | Reptilia | 4/4 | 100 | NR | NR |
| Scincidae | Reptilia | 18/18 | 100 | NR | NR |
| Sphaerodactylidae | Reptilia | 6/6 | 100 | NR | NR |
| Testudinidae | Reptilia | 7/7 | 100 | NR | NR |
| Trionychidae | Reptilia | 5/5 | 100 | NR | NR |
| Typhlopidae | Reptilia | 1/1 | 100 | NR | NR |
| Varanidae | Reptilia | 2/2 | 100 | NR | NR |
| Diverse | Amphibia/Reptilia | 0/0 | 0 | 0.26 | 1.98 |

NR*: Not recorded

ϴ: Percentage of total observed costs

Φ: Percentage of total costs

**
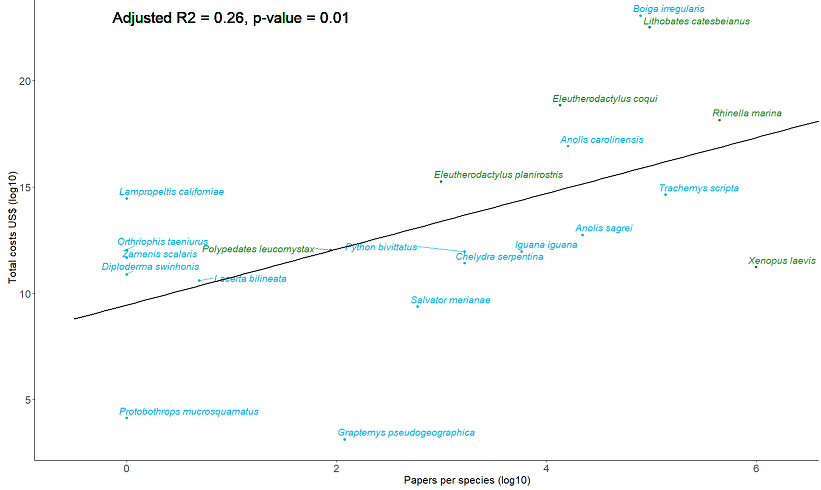
**

Figure S1: Linear regression between the total costs of invasive herpetofauna (y-axis) vs. the number of research documents mentioning each species (x-axis) returned by our search (see main text). Green represents alien amphibians and blue represents alien reptiles.


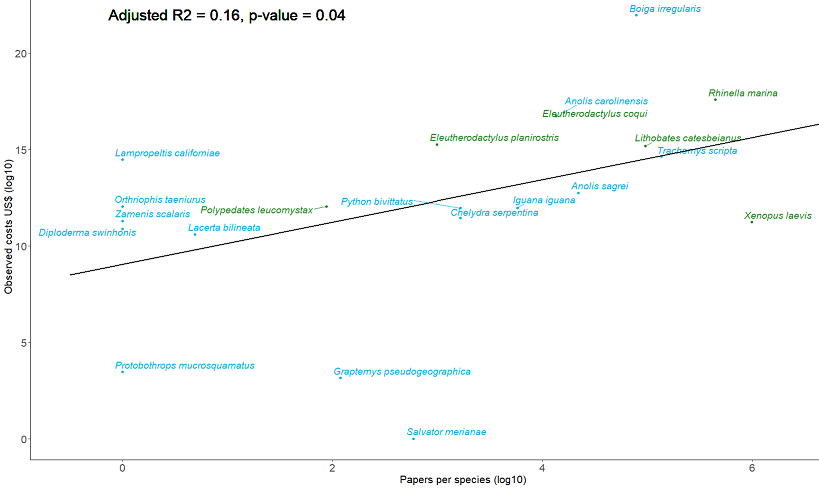


Figure S2: Linear regression between the observed costs of invasive herpetofauna (log-transformed, y-axis) vs. the number of research documents mentioning each species (log-transformed, x-axis) returned by our search (see main text). Green represents alien amphibians and blue represents alien reptiles.
